# Synthesis and Pharmacological Evaluation of Novel Phenyl Sulfonamide Derivatives Designed as Modulators of Pulmonary Inflammatory Response

**DOI:** 10.3390/molecules171214651

**Published:** 2012-12-10

**Authors:** Maria Letícia de Castro Barbosa, Thiago José Figueira Ramos, Ana Carolina Santos de Arantes, Marco Aurélio Martins, Patrícia Machado Rodrigues e Silva, Eliezer J. Barreiro, Lídia Moreira Lima

**Affiliations:** 1 Laboratory of Evaluation and Synthesis of Bioactive Substances (LASSBio®), Federal University of Rio de Janeiro, P.O. Box 68024, 21944-971, Rio de Janeiro, RJ, Brazil; 2 Graduate Program of Chemistry (PGQu), Chemistry Institute, Federal University of Rio de Janeiro, Rio de Janeiro, 21941-909, RJ, Brazil; 3 Laboratory of Inflammation—Oswaldo Cruz Institute, Oswaldo Cruz Foundation (FIOCRUZ), Rio de Janeiro, 21045-900, RJ, Brazil

**Keywords:** inflammation, TNF-α, LASSBio-468, LASSBio-596, phthalimide

## Abstract

In this paper we report the design, synthesis and pharmacological evaluation of a new series of phenyl sulfonamide derivatives **2a**–**h** and **3**–**8** planned by structural modification on the anti-inflammatory prototype LASSBio-468 (**1**). Among the synthesized analogues, the tetrafluorophthalimide LASSBio-1439 (**2e**) stands out showing an *in vitro* anti-TNF-α effect similar to the standard thalidomide. The relevance of tetrafluorination of the phthalimide nucleus was also confirmed by the anti-inflammatory profile of **2e**, through oral administration, in a murine model of pulmonary inflammation. The corresponding tetrafluorocarboxyamide metabolite LASSBio-1454 (**15**), generated from partial hydrolysis of the derivative **2e**, presented a significant *in vitro* effect and a pronounced anti-inflammatory activity *in vivo*.

## 1. Introduction

Inflammation is the body’s response to insults, including trauma, infections and hypersensitivity. In the lung, this response is usually induced by pathogens, toxins, pollutants, irritants and allergens [1]. Normally, the acute inflammatory response is disrupted once the triggering insult is eliminated, the infection is cleared and the damaged tissue is repaired, but if the stimulus is not properly eliminated, the inflammatory process persists and acquires new characteristics, becoming detrimental due to its negative effects on tissue function. In some cases this undesirable inflammatory response results in overt tissue damage [2].

Since the lung is a vital organ for gas exchange, excessive inflammation can be life threatening [1]. Thus, the protective roles and the detrimental potential of the inflammatory response have to be delicately balanced to maintain lung homeostasis [3]. Clinically, acute lung inflammation is present in pneumonia, acute lung injury (ALI) and acute respiratory distress syndrome (ARDS), whereas chronic lung inflammation is represented by asthma and chronic obstructive pulmonary disease (COPD) [1,4,5].

Knowing that inflammation is an important feature of many pulmonary diseases, several strategies have been adopted to interfere with the lung immune response, including the modulation of pro-inflammatory cytokines and the regulation of cellular signal transduction [1].

Tumor necrosis factor alpha (TNF-α) is a crucial cytokine in immunity and inflammation. On the other hand, despite its physiological relevance, it is well-known that the increased biosynthesis and release of TNF-α lead to exacerbation of inflammatory and oxidative responses, which are related to the pathogenesis of a wide range of diseases [6,7]. Among pulmonary diseases, TNF-α is implicated in asthma, COPD, ALI and ARDS [4].

In the context of a research program aimed at the discovery of new anti-inflammatory lead compounds useful in the treatment of airway diseases, Lima and colleagues previously described LASSBio-468 (**1**), a thalidomide analogue with pronounced anti-inflammatory activity in a murine model of acute lung inflammation (ED_50_ = 2.5 mg/kg; i.p.) [8,9,10].

In a continuing effort to identify new anti-inflammatory and immunomodulatory drug candidates, we report in this paper the design, synthesis and pharmacological evaluation of novel phenyl sulfonamide derivatives **2a**–**h** and **3**–**8** planned by structural modifications on the prototype **1**.

The design concept was based on previous reports in literature suggesting the importance of structural modifications on the phthalimide subunit to optimize the anti-TNF-α effect of thalidomide (**9**) and analogues [6,10,11,12,13].

**Scheme 1:**
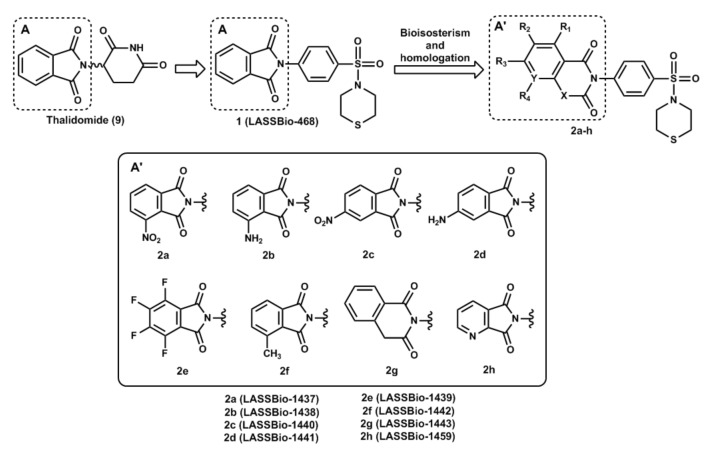
Structural design of the target derivatives **2a**–**h**.

Therefore, the novel compounds **2a**–**f** were designed through the introduction of electron withdrawing or electron donating substituents on the phthalimide core, aiming to study the influence of electronic parameters on the anti-TNF-α activity. Moreover, the homologation strategy and the classical bioisosteric replacement were employed in the design of compounds **2g** and **2h**, respectively (Scheme 1).

**Scheme 2:**
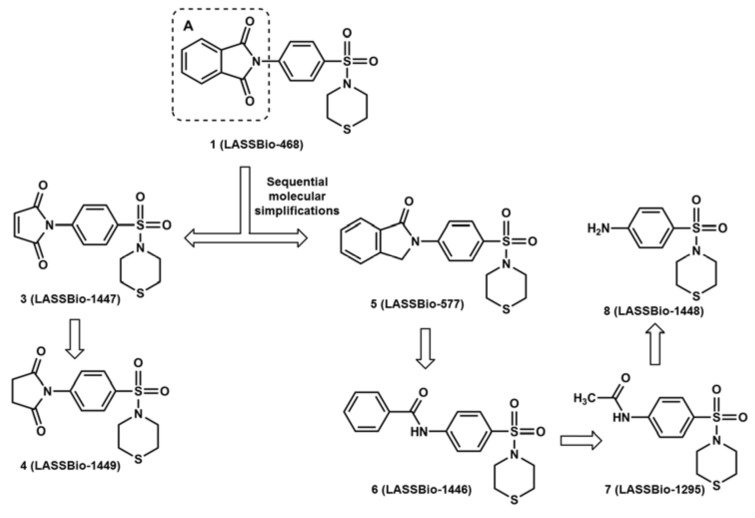
Structural design of the simplified derivatives **3**–**8**.

In an attempt to understand the different pharmacophoric contributions in the phthalimide core to the anti-inflammatory effects of prototype **1**, sequential molecular simplifications were employed in the phthalimide subunit. The simplified derivatives **3**–**8** of LASSBio-468 (**1**) are shown in Scheme 2.

## 2. Results and Discussion

### 2.1. Chemistry

**Scheme 3:**
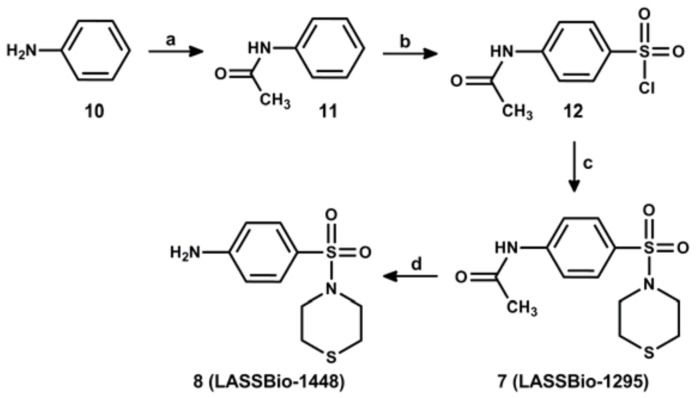
Synthesis of the key intermediate **8** (LASSBio-1448).

The synthesis of the new phenyl sulfonamide analogues **2a**–**h** and **3**–**6** was undertaken employing the aniline LASSBio-1448 (**8**) as a key intermediate. This compound, containing the phenyl sulfonamide fragment of the designed derivatives, was easily obtained in high yield, by the synthetic sequence depicted in Scheme 3, using a classical methodology based on several functional group interconversions.

The acetylation of aniline (**10**) with acetic anhydride provided acetanilide (**11**) in 90% yield [14,15]. The second step in the synthesis of **8** was based on a regioselective electrophilic aromatic substitution employing chlorosulfonic acid, giving **12** in 85% yield [16,17]. With this intermediate in hand, the acetamide LASSBio-1295 (**7**) was synthesized in 65% yield by condensation of the 4-(acetylamino)benzenesulfonyl chloride (**12**) with thiomorpholine [15,18]. The key intermediate **8** was obtained in 93% yield through alkaline hydrolysis of the acetamide **7** (Scheme 3) [19].

**Scheme 4:**
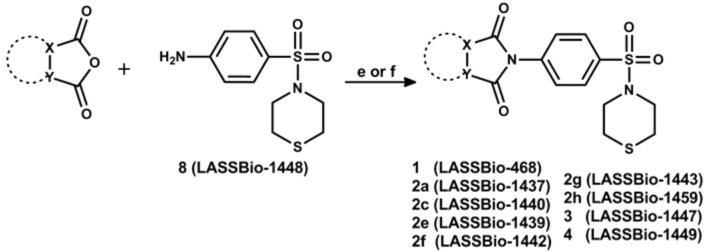
Synthesis of the imide derivatives **2a**, **2c**, **2e**–**h**, **3** and **4**.

Afterwards, the synthesis of the imide core of compounds **2a**, **2c**, **2e**–**h**, **3** and **4** was undertaken employing the condensation of the key intermediate LASSBio-1448 (**8**) with functionalized anhydrides through the methodologies depicted in Scheme 4 [20,21].

**Scheme 5:**
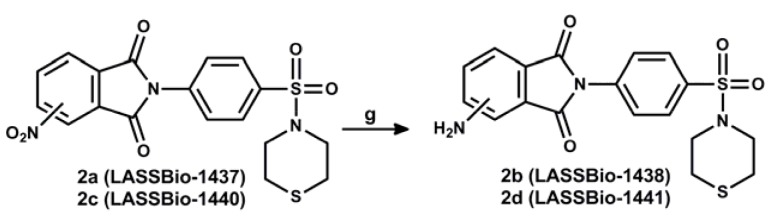
Synthesis of the aminophthalimide analogues **2b** and **2d**.

Then, the aminophthalimide analogues **2b** (LASSBio-1438) and **2d** (LASSBio-1441) were obtained from the nitro derivatives **2a** (LASSBio-1438) and **2c** (LASSBio-1440), respectively, through a functional group interconversion (Scheme 5) [22].

**Scheme 6:**
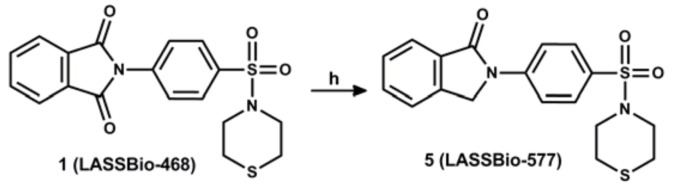
Synthesis of the isoindolinone 5 (LASSBio-577).

**Scheme 7:**
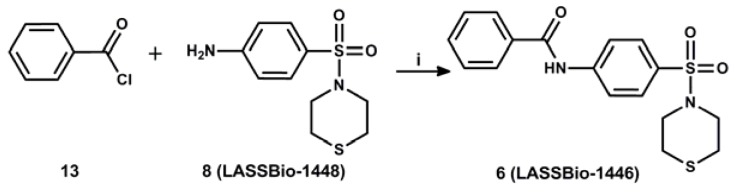
Synthesis of the benzamide **6** (LASSBio-1446).

As described above, the simplified derivatives **7** and **8** were obtained as synthetic intermediates for the preparation of the designed imide analogues of **1** (Scheme 3). Finally, the isoindolinone LASSBio-577 (**5**) was prepared through chemoselective reduction of the phthalimide ring of prototype **1** (LASSBio-468, Scheme 6) [23]; and the benzamide LASSBio-1446 (**6**) was synthesized in 76% yield through the condensation of the key intermediate **8** with benzoyl chloride (**13**, Scheme 7) [24].

### 2.2. Pharmacological Screening

**Figure 1:**
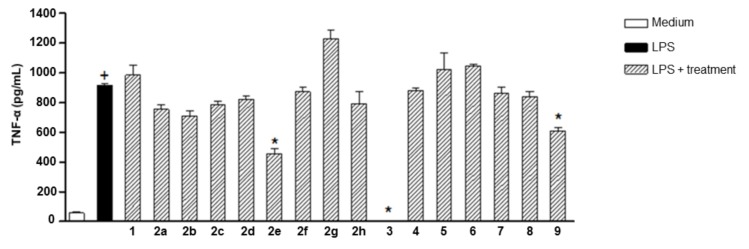
Effect of phenyl sulfonamide derivatives (**1**, **2a**–**h**, **3**–**8**) and standard thalidomide (**9**) on TNF-α production from peritoneal murine macrophages stimulated with LPS *in vitro* (0.1 µg/mL). Screening concentration of 100 μM was used. Values are mean ± SEM. ^+^ *p* < 0.05 compared with the group stimulated with medium; * *p* < 0.05 compared with the group stimulated with LPS; ANOVA followed by Newman-Keuls Student test.

The pharmacological screening of the phenyl sulfonamide derivatives **2a**–**h** and **3**–**8** was based on *in vitro* quantification of TNF-α using an enzyme-linked immuno-sorbent assay (ELISA). These compounds were evaluated for their ability to modulate the production of this pro-inflammatory cytokine in murine macrophages stimulated with lipopolysaccharide (LPS) (Figure 1).

The results depicted in Figure 1 indicate a significant inhibitory effect on murine TNF-α production for the tetrafluorophthalimide derivative LASSBio-1439 (**2e**), which caused 50% inhibition, showing a similar anti-TNF-α effect to the standard thalidomide (**9**), which caused 33% inhibition at a screening concentration of 100 µM.

Interestingly, under these experimental conditions, the phthalimide prototype LASSBio-468 (**1**) did not show any statistically significant inhibitory effect, although previous reports have described its pronounced anti-TNF-α effect *in vivo* after intraperitoneal administration [9].

Taken together, the experimental data demonstrated the relevance of tetrafluorination of the phthalimide ring to optimize the anti-TNF-α profile of the analogue **2e**, confirming the potential of this functionalization to improve the inhibitory effect on this pro-inflammatory cytokine, as previously described [11,13,25,26].

The viability of macrophages was measured using an inverted microscope by the exclusion test with trypan blue, based on the fact that this hydrophilic dye does not cross the plasma membrane of viable cells, in contrast to what occurs in the case of membrane lysis and cell death. Among the synthesized analogues, only the maleimide compound LASSBio-1447 (**3**) has shown cytotoxic activity. This derivative caused a drastic reduction in the percentage of viable cells to 3%, characterizing the cytotoxic profile of **3** at the screening concentration of 100 µM. Therefore, the complete inhibition of TNF-α production observed in the presence of 100 µM of **3** is, in fact, due to 97% death of macrophages.

### 2.3. Chemical Hydrolysis and Plasma Stability Studies

It is well known that the drug thalidomide (**9**) undergoes spontaneous non-enzymatic hydrolytic cleavage at pH 7.4, resulting in partial hydrolysis of all imides present in the structure of **9** and generating the corresponding carboxamide derivatives [27,28]. Considering the structural relatedness of the phthalimide derivatives LASSBio-468 (**1**) and LASSBio-1439 (**2e**) to thalidomide (**9**), we decided to study the chemical (*i.e.*, pH 7.4 phosphate buffered saline-PBS) and the plasma stability of these derivatives. As expected, both compounds have time-dependent lability, generating the corresponding carboxyamide metabolites. However, the tetrafluorophthalimide LASSBio-1439 (**2e**) presented greater kinetics of hydrolysis in plasma and pH 7.4 than the non-fluorinated derivative LASSBio-468 (**1**) (data not shown).

### 2.4. Synthesis and Anti-TNF-α Effect of Carboxyamide Metabolites

Since the hypothesis of hydrolysis of the phthalimide nucleus was confirmed for compounds **1** and **2e**, we decided to perform the synthesis of the corresponding carboxyamide derivatives LASSBio-596 (**14**) and LASSBio-1454 (**15**) to corroborate the supposition that they could be active metabolites *in vivo*.

**Scheme 8:**
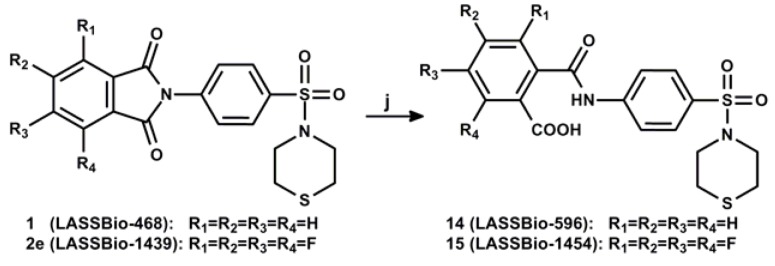
Synthesis of the carboxyamide derivatives **14** and **15**.

The carboxyamide derivatives **14** and **15** were obtained from the phthalimides LASSBio-468 (**1**) and LASSBio-1439 (**2e**), respectively, through alkaline hydrolysis, as previously described for the synthesis of **14** (Scheme 8) [8].

The HPLC and UV analyses of the synthesized compounds LASSBio-596 (**14**) and LASSBio-1454 (**15**) confirmed that they corresponded to the metabolites formed by chemical (pH 7.4) or enzymatic (plasma) hydrolysis of the phthalimides LASSBio-468 (**1**) and LASSBio-1439 (**2e**), respectively.

**Figure 2:**
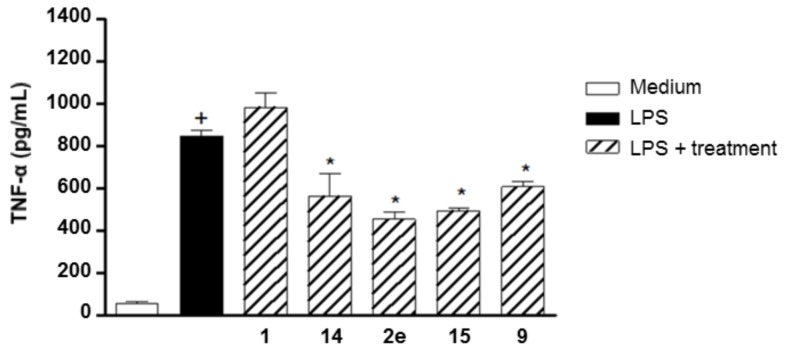
Effect of the phthalimide phenyl sulfonamide derivatives **1** and **2e**, the carboxyamide phenyl sulfonamide derivatives **14** and **15**, and the standard thalidomide (**9**) on TNF-α production from peritoneal murine macrophages stimulated with LPS *in vitro* (0.1 µg/mL). Screening concentration of 100 μM was used. Values are mean ± SEM. ^+^ *p* < 0.05 compared with the group stimulated with medium; * *p* < 0.05 compared with the group stimulated with LPS; ANOVA followed by Newman-Keuls Student test.

The carboxyamide metabolites **14** and **15** were also evaluated for their ability to modulate the production of TNF-α in murine macrophages stimulated with lipopolysaccharide (LPS), comparing the results to those obtained for the phthalimide precursors **1** and **2e** (Figure 2).

The tetrafluorinated carboxyamide LASSBio-1454 (**15**) showed an anti-TNF-α effect *in vitro* (42% inhibition; 100 μM) similar to that observed for the tetrafluorophthalimide **2e** (46% inhibition; 100 μM), indicating that **15** could be partly responsible for the anti-TNF-α activity observed for **2e**.

Additionally, the carboxyamide LASSBio-596 (**14**), probably generated *in vivo* as a metabolite of the prototype **1**, showed significant anti-TNF-α effect (34% inhibition; 100 μM). This fact can justify why the prototype **1** did not demonstrate any statistically significant TNF-α inhibition *in vitro* in the screening assay, although it had shown a pronounced anti-TNF-α effect *in vivo* after intraperitoneal administration [9].

### 2.5. Pharmacological Evaluation in Acute Lung Inflammation

**Figure 3:**
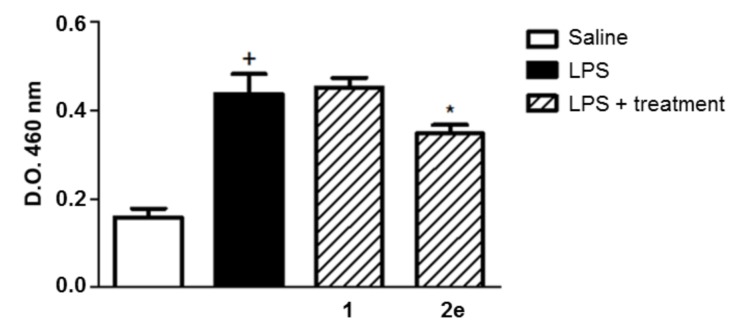
Effect of the phthalimide derivatives **1** and **2e** (50 mg/kg; p.o.) on *pulmonary neutrophil infiltration* in a murine model of acute lung inflammation induced by LPS (25 μg/animal, i.n.). Values are mean ± SEM from six animals. *^+^ p <* 0.05 as compared with saline group; * *p* < 0.05 as compared with the group stimulated with LPS; ANOVA followed by Newman-Keuls Student test.

**Figure 4:**
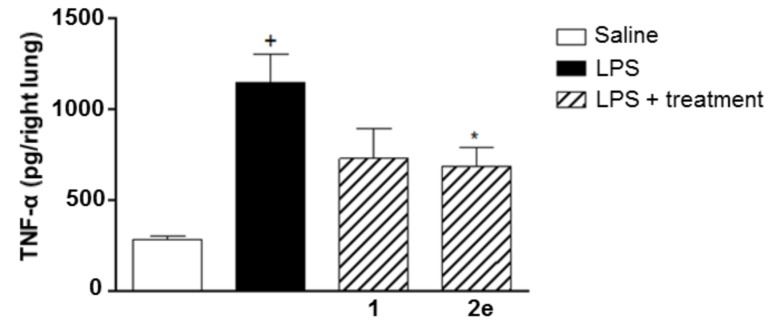
Effect of the phthalimide derivatives **1** and **2e** (50 mg/kg; p.o.) on tissue TNF-α production in a murine model of acute lung inflammation induced by LPS (25 μg/animal, i.n.). Values are mean ± SEM from six animals. *^+^ p <* 0.05 as compared with saline group; * *p* < 0.05 as compared with the group stimulated with LPS; ANOVA followed by Newman-Keuls Student test.

In view of the previously published results describing **1** as an anti-inflammatory prototype with pronounced effect after intraperitoneal administration [8]; we decided to evaluate the oral anti-inflammatory effect of the lead-compound **1** and its tetrafluorophthalimide analogue **2e** in a murine model of pulmonary inflammation induced by LPS (Figure 3 and Figure 4). The results depicted in Figure 3 and Figure 4 demonstrated once again, now in an *in vivo* model, the relevance of the tetra-fluorination of the phthalimide ring to the optimization of anti-inflammatory and anti-TNF-α profiles, once the prototype **1** (LASSBio-468) was orally inactive at the dose of 50 mg/kg, while its tetrafluorophthalimide analogue **2e** significantly inhibited the infiltration of neutrophils (32% inhibition; 50 mg/kg; p.o.) and the production of TNF-α (37% inhibition; 50 mg/kg, p.o.) in the lung tissue. 

Considering the high rate of hydrolysis detected *in vitro* for the tetrafluorophthalimide nucleus of **2e**, the anti-inflammatory and anti-TNF-α effects observed for this compound *in vivo* are probably due to the corresponding carboxyamide metabolite LASSBio-1454 (**15**), suggesting that **15** would be an active metabolite of **2e**. Aiming to verify if the oral administration of the carboxyamide metabolites **14** or **15** could also result in an anti-inflammatory effect, these compounds were evaluated in the same animal model of acute lung inflammation. 

Both compounds were orally inactive at the doses of 50 mg/kg or 100 mg/kg. This result can be associated to a limited oral bioavailability of the carboxyamide derivatives **14** and **15**. In fact, previous results demonstrated that **14** has an oral bioavailability of 3.6% in Wistar rats [29].

**Figure 5:**
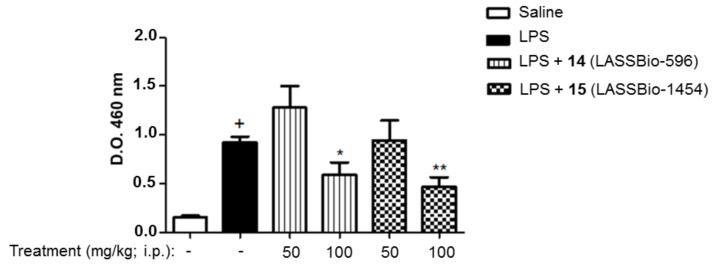
Effect of the carboxyamide derivatives **14** and **15** (50 and 100 mg/kg; i.p.) on *tissue neutrophil infiltration* in a murine model of acute lung inflammation induced by LPS (25 μg/animal, i.n.). Values are mean ± SEM from six animals. *^+^ p <* 0.05 as compared with saline group; * *p* < 0.05 as compared with the group stimulated with LPS; ** *p* < 0.01 as compared with the group stimulated with LPS; ANOVA followed by Newman-Keuls Student test.

**Figure 6:**
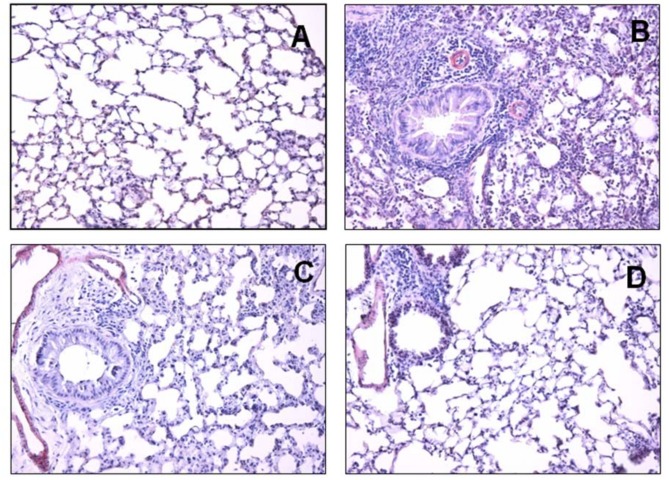
Light photomicrographs of lung tissue sections from mice stimulated with LPS (25 μg/animal, i.n.). (**A**) saline; (**B**) LPS; (**C**) LPS + **14**; (D) LPS + **15**. The effect of carboxyamide derivatives **14** and **15** (100 mg/kg; i.p.) was evaluated 24 h after LPS stimulation. Hetamoxylin & eosin staining. (200×).

Thus, to confirm this hypothesis, the anti-inflammatory activity of the carboxyamides **14** and **15** was further evaluated in the same model by intraperitoneal administration (Figure 5). After changing the drug administration route, the tetrafluoroderivative **15** showed a higher inhibitory effect for controlling the infiltration of neutrophils into the lung tissue (59% inhibition; 100 mg/kg; i.p.), when compared to the non-fluorinated analogue **14** (43% inhibition; 100 mg/kg; i.p.). As demonstrated in Figure 6, the intranasal instillation of LPS induced a pronounced inflammatory response characterized by the presence of inflammatory cells in the lung (Figure 6B), that was interestingly reduced by intraperitoneal treatment with **14** (Figure 6C) and **15** (Figure 6D).

**Figure 7:**
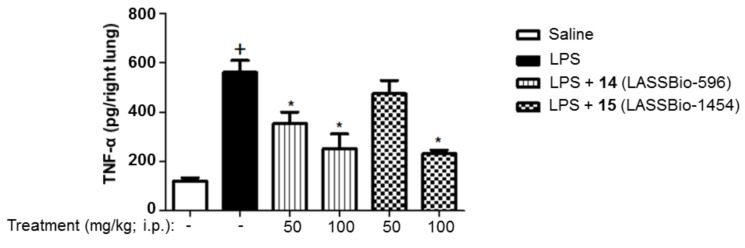
Effect of the carboxyamide derivatives **14** and **15** (50 and 100 mg/kg; i.p.) on TNF-α production in the lung tissue of mice stimulated with LPS (25 μg/animal, i.n.). Values are mean ± SEM from six animals. *^+^*p* <* 0.05 as compared with saline group; * *p* < 0.05 as compared with the group stimulated with LPS; ANOVA followed by Newman-Keuls Student test.

The ability of compounds **14** and **15** to inhibit TNF-α production *in vivo* in the lung tissue of mice stimulated with LPS was also determined. These compounds have shown similar anti-TNF-α effects *in vivo* at the dose of 100 mg/kg after intraperitoneal administration (55% and 59% inhibition, respectively). However, only compound **14** was able to inhibit the production of this cytokine at the lower dose of 50 mg/kg (i.p.; 37% inhibition) (Figure 7).

**Figure 8:**
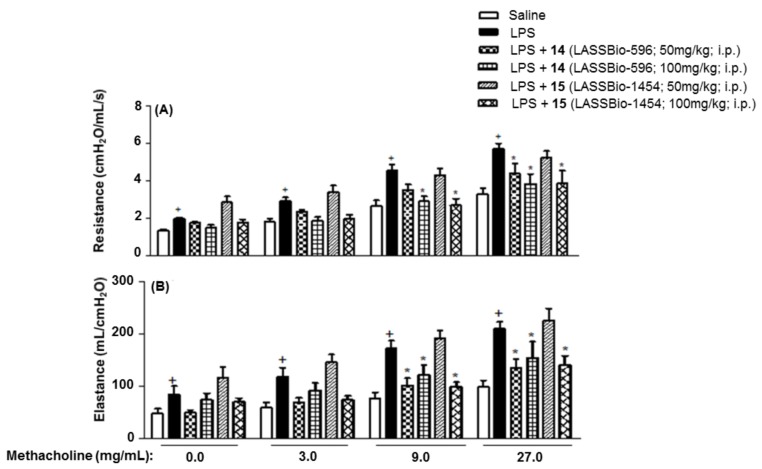
Effect of the carboxyamide derivatives **14** and **15** (50 and 100 mg/kg; i.p.) on methacholine-induced increase of lung resistance (**A**) and elastance (**B**) in mice stimulated with LPS (25 μg/animal, i.n.). Values are mean ± SEM from six animals. *^+^*p* <* 0.05 as compared with saline group; * *p* < 0.05 as compared with the group stimulated with LPS; ANOVA followed by Newman-Keuls Student test.

Furthermore, the carboxyamide analogue LASSBio-596 (**14**) improved the parameters of lung elastance and resistance in these animals at the doses of 50 mg/kg and 100 mg/kg, effect that was observed for the fluorinated analogue LASSBio-1454 (**15**) only at the dose of 100 mg/kg (Figure 8).

In view of the presented results, the carboxyamide derivative **15** (LASSBio-1454) can be considered a new anti-inflammatory prototype able to improve the parameters of lung elastance and resistance and to reduce the inflammatory cell migration and TNF-α release during the lung inflammation process.

## 3. Experimental

### 3.1. General

Reactions were routinely monitored by thin-layer chromatography (TLC) in silica gel (F245 Merck plates) and the products visualized with iodine or ultraviolet lamp (254 and 365 nm). ^1^H-, ^19^F- and ^13^C- NMR spectra were determined in DMSO-*d_6_*, DMF-*d_7_* or CDCl_3_ solutions using a Bruker AC-200 or a Varian UNITY-300 spectrometers. The chemical shifts are given in parts per million (*δ*) from tetramethylsilane as internal standard, and coupling constant values (*J*) are given in Hz. Signal multiplicities are represented by: s (singlet), d (doublet), t (triplet), q (quadruplet), qu (quintuplet), m (multiplet) and br (broad signal). Infrared (IR) spectra were obtained using an ABB FTLA2000-100 IR spectrometer. Samples were examined as potassium bromide (KBr) disks. Elemental microanalyses were obtained on an Elemental Analyzer (Flash EA 1112 Series, Thermo Scientific) from vacuum-dried samples. The analytical results for C, H, and N were within ± 0.4% of the theoretical values. Melting points were determined using a Quimis instrument and are uncorrected. All described products have shown-NMR spectra according to the assigned structures. All organic solutions were dried over anhydrous sodium sulphate and all organic solvents were removed under reduced pressure in rotatory evaporator.

*N-Phenylacetamide* (**11**). A solution of anhydrous sodium acetate (2.1 g, 25.6 mmol) in glacial acetic acid (8.0 mL, 139.9 mmol) was prepared. Aniline (**10**, 8.0 mL, 87.8 mmol) was slowly added, followed by acetic anhydride (8.5 mL, 90.1 mmol). The reaction mixture was stirred for 30 min at room temperature and the end of the reaction was checked by TLC. After cooling, the *N*-phenylacetamide (**11**) was filtered through a Büchner funnel, washed twice with 200 mL of water and obtained as a white shiny powder, 90% yield, mp 116 °C. The melting point, ^1^H-NMR and IR data for compound **11** are in agreement with previous reports [14,30].

*4-(Acetylamino)benzenesulfonyl chloride* (**12**). Chlorosulfonic acid (5.2 mL, 78.27 mmol) was slowly added to *N*-phenylacetamide (11, 2.0 g, 14.80 mmol). The resulting mixture was stirred and heated at 60 °C for 30 min. After cooling, the reaction medium was poured into a water and ice mixture. Then, the 4-(acetylamino)benzenesulfonyl chloride (**12**) was filtered through a Büchner funnel, washed twice with 100 mL of water and was obtained as a white powder, 85% yield, mp 143–148 °C. The melting point and IR data for compound **12** are in agreement with previous reports [16,17].

*N-[4-(Thiomorpholin-4-ylsulfonyl)phenyl]acetamide* (LASSBio-1295, **7**). Thiomorpholine (0.5 mL, 5.0 mmol) was added to a solution of the sulfonylchloride derivative **12** (500 mg, 2.14 mmol) in methylene chloride (50 mL). The reaction mixture was stirred for 30 min at room temperature and the end of the reaction was checked by TLC. The sulfonamide **7** was isolated by addition of 50 mL of methylene chloride and extraction, washing the organic layer with 10% aq HCl and brine. The organic layer was then dried over anhydrous Na_2_SO_4_, filtered and concentrated at reduced pressure to give the title compound as a yellow powder, 65% yield, mp 207–209 °C. The melting point, ^1^H-NMR, ^13^C-NMR and IR data for compound **7** are in agreement with previous reports [15]. ^1^H-NMR (200 MHz, DMSO-*d_6_*, TMS) δ (ppm): 2.09 (s, 3H, COCH_3_); 2.65 (t, 4H, *J* = 5.0 Hz, H2' and H6'); 3.16 (t, 4H, *J* = 5.0 Hz, H3' and H5'); 7.67 (d, 2H, *J* = 8.9 Hz, H2 and H6); 7.82 (d, 2H, *J* = 8.9 Hz, H3 and H5); 10.40 (s, 1H, CONH). ^13^C-NMR (50 MHz, DMSO-*d_6_*, TMS) δ (ppm): 24.2 (COCH_3_); 26.4 (C-2' and C-6'); 47.8 (C-3' and C-5'); 118.8 (C-2 and C-6); 128.5 (C-3 and C-3); 129.5 (C-4); 143.5 (C-1); 169.2 (C=O). IR (υ_max_, KBr) υ (cm^−1^): 3352, 1702, 1321, 1153, 835. *Anal*. Calcd. for C_12_H_16_N_2_O_3_S_2_: C, 47.98; H, 5.37; N, 9.33. Found: C, 47.84; H, 5.33; N, 9.24.

*4-(Thiomorpholin-4-ylsulfonyl)aniline* (LASSBio-1448, **8**). A solution of potassium hydroxide (4.67 g, 83.33 mmol) in water (5 mL) was slowly added to a solution of the acetamide **7** (5 g, 16.67 mmol) in methanol (25 mL). The reaction mixture was stirred and heated at 60 °C for 3 h and the end of the reaction was checked by TLC. The aniline derivative **8** was isolated by concentration at reduced pressure, followed by filtration of obtained precipitate through a Büchner funnel. Purification was performed through recrystallization from ethanol, giving the title compound as yellow needles, 93% yield, mp 179–181 °C. The melting point for compound **8** is in agreement with previous reports [31]. ^1^H-NMR (200 MHz, CDCl_3_, TMS) δ (ppm): 2.71 (br, 4H, H2' and H6'); 3.29 (br, 4H, H3' and H5'); 4.15 (br, 2H, NH_2_); 6.69 (d, 2H, *J* = 8.3 Hz, H3 and H5); 7.50 (d, 2H, *J* = 8.3 Hz, H2 and H6). ^13^C-NMR (50 MHz, CDCl_3_, TMS) δ (ppm): 27.4 (C-2' and C-6'); 48.0 (C-3' and C-5'); 114.2 (C-3 e C-5); 124.7 (C-4); 129.7 (C-2 and C-6); 150.9 (C-1). IR (υ_max_, KBr) υ (cm^−1^): 3454, 3368, 1635, 1314, 1153, 827. *Anal*. Calcd. for C_10_H_14_N_2_O_2_S_2_: C, 46.49; H, 5.46; N, 10.84. Found: C, 46.65; H, 5.40; N, 10.70.

*1-(4-(Thiomorpholinosulfonyl)phenyl)-1H-pyrrole-2,5-dione* (LASSBio-1447, **3**). In a 25 mL flask equipped with a reflux condenser, the reaction mixture containing the key intermediate LASSBio-1448 (**8**, 500 mg, 1.94 mmol), maleic anhydride (196.12 mg, 2.00 mmol) and glacial acetic acid (8.0 mL, 140 mmol) was stirred and heated at 140 °C for 3 h. The end of the reaction was observed by TLC and the reaction medium was then cooled in an ice bath. The obtained precipitate was filtered through a Büchner funnel and purified in a silica gel chromatographic column using CH_2_Cl_2_ 100% as eluent. The title compound was isolated as yellow crystals, 73% yield, mp 184 °C. ^1^H-NMR (200 MHz, DMSO-*d_6_*, TMS) δ (ppm): 2.68 (br, 4H, H2'' and H6''); 3.24 (br, 4H, H3'' e H5''); 7.25 (s, 2H, H4 and H5); 7.66 (d, 2H, *J* = 8.2 Hz, H3' and H5'); 7.88 (d, 2H, *J* = 8.2 Hz, H2' and H6'). ^13^C-NMR (50 MHz, DMSO-*d_6_*, TMS) δ (ppm): 26.4 (C2'' and C6''); 47.8 (C3'' and C5''); 126.7 (C-3' and C-5'); 128.0 (C-2' and C-6'); 134.7 (C-4'); 135.0 (C-4 and C-5); 135.8 (C-1'); 169.4 (C-1 and C-3). IR (υ_max_, KBr) υ (cm^−1^): 3099, 2926, 2849, 1711, 1376, 1161, 968, 838. *Anal*. Calcd. for C_14_H_14_N_2_O_4_S_2_: C, 49.69; H, 4.17; N, 8.28. Found: C, 49.64; H, 4.09; N, 8.28.

#### 3.1.1. General Procedure for Synthesis of Imide *N*-phenyl Sulfonamide Derivatives

In a 10 mL flask equipped with a reflux condenser, the key intermediate LASSBio-1448 (**8**, 100 mg, 0.39 mmol; mp 179–181 °C) was heated until complete melting. Then, the functionalized anhydrides (0.80 mmol) were slowly added and the reaction mixture was stirred and heated at 180 °C until the end of reaction (as indicated by TLC, 1–4 h). The reaction medium was cooled down to room temperature, distilled water was added and the obtained precipitate was filtered through a Büchner funnel and washed with ethanol.

*2-(4-(Thiomorpholinosulfonyl)phenyl)isoindoline-1,3-dione* (LASSBio-468, **1**). The title compound was obtained by condensation of **8** with phthalic anhydride as white crystals, 80% yield after recrystallization from ethanol, mp 190–192 °C. The melting point, ^1^H-NMR, ^13^C-NMR and IR data for compound **1** are in agreement with previous reports [8]. ^1^H-NMR (200 MHz, DMSO-*d_6_*, TMS) δ (ppm): 2.69 (t, 4H, *J =* 5.0 Hz, H2'' and H6''); 3.27 (t, 4H, *J =* 5.0 Hz, H3'' and H5''); 7.78 (d, 2H, *J* = 8.5 Hz, H3' and H5'); 7.97 (m, 6H, H2', H6', H4, H5, H6 and H7). ^13^C-NMR (50 MHz, DMSO-*d_6_*, TMS) δ (ppm): 26.4 (C2'' and C6''); 47.8 (C3'' and C5''); 123.5 (C-4 and C-7); 127.6 (C-3' and C-5'); 127.9 (C-2' and C-6'); 131.4 (C-3a and C-7a); 134.9 (C-5 and C-6); 135.1 (C-4'); 136.0 (C-1'); 166.5 (C-1 and C-3). IR (υ_max_, KBr) υ (cm^−1^): 2908, 2855, 1789, 1717, 1370, 1169, 834, 743. *Anal*. Calcd. for C_18_H_16_N_2_O_4_S_2_: C, 55.65; H, 4.15; N, 7.21. Found: C, 55.58; H, 4.22; N, 7.18.

*4-Nitro-2-(4-(thiomorpholinosulfonyl)phenyl)isoindoline-1,3-dione* (LASSBio-1437, **2a**). The title compound was obtained by condensation of **8** with 3-nitrophthalic anhydride as yellow crystals, 95% yield after recrystallization from ethanol, mp > 250 °C. ^1^H-NMR (200 MHz, DMSO-*d_6_*, TMS) δ (ppm): 2.69 (br, 4H, H2'' and H6''); 3.27 (br, 4H, H3'' and H5''); 7.75 (d, 2H, *J* = 8.0 Hz, H3' and H5'); 7.94 (d, 2H, *J* = 8.0 Hz, H2' and H6'); 8.14 (dd, 1H, *J* = 7.5 Hz and 7.9 Hz, H6); 8.30 (d, 1H, *J* = 7.5 Hz, H7); 8.37 (d, 1H, *J* = 7.9 Hz, H5). ^13^C-NMR (50 MHz, DMSO-*d_6_*, TMS) δ (ppm): 26.5 (C2'' and C6''); 47.8 (C3'' and C5''); 122.8 (C-3a); 127.2 (C-5); 127.9 (C-3' and C-5'); 128.0 (C-2' and C-6'); 128.6 (C-7); 133.5 (C-7a); 135.6 (C-4'); 135.8 (C-1'); 136.6 (C-6); 144.6 (C-4); 162.2 (C-3); 164.7 (C-1). IR (υ_max_, KBr) υ (cm^−1^): 3109, 2920, 2855, 1789, 1731, 1537, 1370, 1337, 1169, 833, 770, 709. *Anal*. Calcd. for C_18_H_15_N_3_O_6_S_2_: C, 49.88; H, 3.49; N, 9.69. Found: C, 49.83; H, 3.42; N, 9.58.

*5-Nitro-2-(4-(thiomorpholinosulfonyl)phenyl)isoindoline-1,3-dione* (LASSBio-1440, **2c**). The title compound was obtained by condensation of **8** with 4-nitrophthalic anhydride as yellow crystals, 93% yield after recrystallization from ethanol, mp > 250 °C. ^1^H-NMR (200 MHz, DMSO-*d_6_*, TMS) δ (ppm): 2.70 (br, 4H, H2'' and H6''); 3.28 (br, 4H, H3'' and H5''); 7.79 (d, 2H, *J* = 8.5 Hz, H3' and H5'); 7.96 (d, 2H, *J* = 8.5 Hz, H2' and H6'); 8.26 (d, 1H, *J* = 8.2 Hz, H7); 8.63 (s, 1H, H4); 8.70 (d, 1H, *J* = 8.2 Hz, H6). ^13^C-NMR (50 MHz, DMSO-*d_6_*, TMS) δ (ppm): 26.5 (C2'' and C6''); 47.9 (C3'' and C5''); 118.4 (C-4); 125.1 (C-7); 127.7 (C-3' and C-5'); 128.1 (C-2' and C-6'); 130.0 (C-6); 133.0 (C-3a); 135.6 (C-4'); 135.7 (C-1'); 136.2 (C-7a); 151.7 (C-5); 164.8 (C-3); 165.1 (C-1). IR (υ_max_, KBr) υ (cm^−1^): 3106, 2925, 2850, 1780, 1723, 1535, 1379, 1339, 1164, 823. *Anal*. Calcd. for C_18_H_15_N_3_O_6_S_2_: C, 49.88; H, 3.49; N, 9.69. Found: C, 50.10; H, 3.47; N, 9.63.

*4,5,6,7-Tetrafluoro-2-(4-(thiomorpholinosulfonyl)phenyl) isoindoline-1,3-dione* (LASSBio-1439, **2e**). The title compound was obtained by condensation of **8** with 3,4,5,6-tetrafluorophthalic anhydride as a white shiny powder, 93% yield after purification in a silica gel chromatographic column using CH_2_Cl_2_ 100% as eluent, mp 195–197 °C. The ^13^C-NMR data are not described for this compound, as the spectrum obtained employing a conventional proton decoupling experiment was very cumbersome to interpret unequivocally. It’s well known that ^13^C-NMR spectra for polyfluorinated compounds are difficult to obtain for several reasons. First of all, in the absence of directly bound protons, lack of nuclear Overhauser enhancement and slow relaxation rates can give weaker signals. On the other hand, multicoupling to fluorine ^19^F nucleus (^1^J, ^2^J and ^3^J) reduces even more the intensity of them and gives a complex spectrum [32]. ^1^H-NMR (300 MHz, DMF-*d_7_*, TMS) δ (ppm): 2.76 (t, 4H, *J =* 5.0 Hz, H2'' and H6''); 3.36 (t, 4H, *J =* 5.0 Hz, H3'' and H5''); 7.89 (d, 2H, *J* = 8.5 Hz, H3' and H5'); 8.05 (d, 2H, *J* = 8.5 Hz, H2' and H6’). ^19^F-NMR (282 MHz, DMF-*d_7_*) δ (ppm): −145.3 (dd, 2F, I*J*I = 9.6 Hz and 20.6 Hz, F-5 and F-6); −139.6 (dd, 2F, I*J*I = 9.6 Hz and 20.6 Hz, F-4 and F-7). IR (υ_max_, KBr) υ (cm^−1^): 3122, 2900, 1788, 1727, 1408, 1344, 1161, 824. *Anal*. Calcd. for C_18_H_12_F_4_N_2_O_4_S_2_: C, 46.96; H, 2.63; N, 6.08. Found: C, 47.07; H, 2.61; N, 6.01.

*4-Methyl-2-(4-(thiomorpholinosulfonyl)phenyl)isoindoline-1,3-dione* (LASSBio-1442, **2f**). The title compound was obtained by condensation of **8** with 3-methylphthalic anhydride as beige shiny crystals, 77% yield after recrystallization from ethanol, mp 173 °C. ^1^H-NMR (200 MHz, DMSO-*d_6_*, TMS) δ (ppm): 2.68 (br, 7H, CH_3_, H2'' and H6''); 3.27 (br, 4H, H3'' and H5''); 7.75 (m, 5H, H5, H6, H7, H3' and H5'); 7.92 (d, 2H, *J* = 8.6 Hz, H2' and H6’). ^13^C-NMR (50 MHz, DMSO-*d_6_*, TMS) δ (ppm): 17.2 (CH_3_); 26.5 (C2'' and C6''); 47.9 (C3'' and C5''); 121.3 (3a); 127.7 (C-3' and C-5'); 127.9 (C-2' and C-6'); 128.1 (C-4); 131.9 (C-7a); 134.4 (C-4'); 135.1 (C-1'); 136.1 (C-7); 137.0 (C-5); 137.8 (C-6); 166.4 (C-3); 167.1 (C-1). IR (υ_max_, KBr) υ (cm^−1^): 3044, 2919, 2851, 1774, 1724, 1371, 1157, 834, 815, 716. *Anal*. Calcd. for C_19_H_18_N_2_O_4_S_2_: C, 56.70; H, 4.51; N, 6.96. Found: C, 56.55; H, 4.48; N, 6.82.

*2-(4-(Thiomorpholinosulfonyl)phenyl)isoquinoline-1,3(2H,4H)-dione* (LASSBio-1443, **2g**). The title compound was obtained by condensation of **8** with homophthalic anhydride as yellow shiny crystals, 60% yield after recrystallization from ethanol, mp > 250 °C. ^1^H-NMR (200 MHz, DMSO-*d_6_*, TMS) δ (ppm): 2.70 (br, 4H, H2'' and H6''); 3.28 (br, 4H, H3'' and H5''); 4.30 (s, 2H, H4); 7.51 (m, 4H, H5, H6, H3' and H5'); 7.73 (dd, 1H, *J* = 7.2 Hz and 7.6 Hz, H7); 7.88 (d, 2H, *J* = 8.4 Hz, H2' and H6'); 8.07 (d, 1H, *J* = 7.6 Hz, H8). ^13^C-NMR (50 MHz, DMSO-*d_6_*, TMS) δ (ppm): 26.5 (C2'' and C6''); 36.7 (C-4); 47.9 (C3'' and C5''); 125.1 (C-8a); 127.4 (C-5); 127.7 (C-7); 127.9 (C-3' and C-5'); 128.1 (C-8); 130.4 (C-2' and C-6'); 133.9 (C-6); 135.8 (C-4a); 136.0 (C-4'); 140.4 (C-1'); 164.7 (C-1); 169.9 (C-3). IR (υ_max_, KBr) υ (cm^−1^): 3100, 2921, 2854, 1724, 1678, 1368, 1160, 819, 752. *Anal*. Calcd. for C_19_H_18_N_2_O_4_S_2_: C, 56.70; H, 4.51; N, 6.96. Found: C, 56.66; H, 4.54; N, 6.92.

*6-(4-(Thiomorpholinosulfonyl)phenyl)-5H-pyrrolo[3,4-b]pyridine-5,7(6H)-dione* (LASSBio-1459, **2h**). The title compound was obtained by condensation of **8** with pyridine 2,3-dicarboxylic anhydride as yellow crystals, 50% yield after recrystallization from ethanol, mp > 250 °C. ^1^H-NMR (200 MHz, CDCl_3_, TMS) δ (ppm): 2.74 (t, 4H, *J* = 5.0 Hz, H2'' and H6''), 3.42 (t, 4H, *J* = 5.0 Hz, H3'' and H5''); 7.76 (m, 3H, H6, H3' and H5'); 7.91 (d, 2H, *J* = 8.7 Hz, H2' and H6'), 8.32 (d, 1H, *J* = 6.4 Hz, H7); 9.10 (d, 1H, *J =* 3.3 Hz, H5). ^13^C-NMR (50 MHz, CDCl_3_, TMS) δ (ppm): 27.5 (C2'' and C6''); 48.0 (C3'' and C5''); 126.6 (C-3' and C-5'); 127.0 (C-7a); 128.3 (C-6); 128.5 (C-2' and C-6'); 132.1 (C-7); 135.5 (C-4'); 136.7 (C-1'); 151.0 (C-3a); 156.5 (C-5); 164.62 (C-3); 164.65 (C-1). IR (υ_max_, KBr) υ (cm^−1^): 3073, 2931, 2847, 1753, 1730, 1375, 1333, 1172, 838, 806, 700. *Anal*. Calcd. for C_17_H_15_N_3_O_4_S_2_: C, 52.43; H, 3.88; N, 10.79. Found: C, 52.50; H, 3.76; N, 10.87.

*1-(4-(Thiomorpholinosulfonyl)phenyl)pyrrolidine-2,5-dione* (LASSBio-1449, **4**). The title compound was obtained by condensation of **8** with succinic anhydride as a white shiny powder, 85% yield after purification in a silica gel chromatographic column using CH_2_Cl_2_ 100% as eluent, mp > 250 °C. ^1^H-NMR (200 MHz, DMSO-*d_6_*, TMS) δ (ppm): 2.68 (t, 4H, *J* = 4.5 Hz, H2'' and H6''); 2.81 (s, 4H, H4 and H5); 3.25 (t, 4H, *J* = 4.5 Hz, H3'' and H5''); 7.58 (d, 2H, *J* = 8.5 Hz, H3' and H5'); 7.89 (d, 2H, *J* = 8.5 Hz, H2' and H6'). ^13^C-NMR (50 MHz, DMSO-*d_6_*, TMS) δ (ppm): 26.4 (C2'' and C6''); 28.6 (C-4 and C-5); 47.8 (C3'' and C5''); 127.8 (C-3' and C-5'); 127.9 (C-2' and C-6'); 135.5 (C-4'); 136.8 (C-1'); 176.6 (C-1 and C-3). IR (υ_max_, KBr) υ (cm^−1^): 2916, 2858, 1781, 1706, 1393, 1160, 826. *Anal*. Calcd. for C_14_H_16_N_2_O_4_S_2_: C, 49.40; H, 4.74; N, 8.23. Found: C, 49.27; H, 4.68; N, 8.17.

#### 3.1.2. General Procedure for Synthesis of Aminophthalimide *N*-phenyl Sulfonamide Derivatives

In a 50 mL flask equipped with a reflux condenser, a reaction mixture containing the nitrophthalimide derivative (0.46 mmol), metallic iron dust (146 mg, 2.6 mmol), ammonium chloride (73.8 mg, 1.38 mmol), ethanol (12 mL) and distilled water (6 mL) was stirred and heated at 78 °C for 2 h. The end of the reaction was checked by TLC. The product was isolated by filtration on Celite^®^ followed by washing with methylene chloride. Subsequently, the organic extract obtained was washed with brine, dried over anhydrous Na_2_SO_4_, filtered and concentrated at reduced pressure. The residue was purified in a silica gel chromatographic column using MeOH:CH_2_Cl_2_ 1% as eluent.

*4-Amino-2-(4-(thiomorpholinosulfonyl)phenyl)isoindoline-1,3-dione* (LASSBio-1438, **2b**). The title compound was obtained from the nitrophthalimide precursor LASSBio-1437 (**2a**) as yellow crystals, 61% yield after purification by chromatographic column, mp 195–197 °C. ^1^H-NMR (200 MHz, DMSO-*d_6_*, TMS) δ (ppm): 2.69 (t, 4H, *J* = 5.0 Hz, H2'' and H6''); 3.26 (t, 4H, *J* = 5.0 Hz, H3'' and H5''); 6.62 (s, 2H, NH_2_); 7.08 (d, 1H, *J* = 8.4 Hz, H5); 7.10 (d, 1H, *J* = 7.1 Hz, H7); 7.52 (dd, 1H, *J* = 7.1 Hz and 8.4 Hz, H6); 7.75 (d, 2H, *J* = 8.7 Hz, H3' and H5’); 7.89 (d, 2H, *J* = 8.7 Hz, H2' and H6'). ^13^C-NMR (50 MHz, DMSO-*d_6_*, TMS) δ (ppm): 26.5 (C2'' and C6''); 47.8 (C3'' and C5''); 108.6 (C-7); 111.3 (C-5); 121.8 (C-3a); 127.3 (C-3' and C-5'); 127.9 (C-2' and C-6'); 132.0 (C-7a); 134.7 (C-6); 135.7 (C-4'); 136.3 (C-1'); 147.1 (C-4); 166.6 (C-3); 167.8 (C-1). IR (υ_max_, KBr) υ (cm^−1^): 3464, 3365, 2927, 2849, 1768, 1715, 1637, 1370, 1164, 834, 814, 719. *Anal*. Calcd. for C_18_H_17_N_3_O_4_S_2_: C, 53.58; H, 4.25; N, 10.41. Found: C, 53.47; H, 4.28; N, 10.39.

*5-Amino-2-(4-(thiomorpholinosulfonyl)phenyl)isoindoline-1,3-dione* (LASSBio-1441, **2d**). The title compound was obtained from the nitrophthalimide precursor **2c** as yellow crystals, 57% yield after purification by chromatographic column, mp 230°C. ^1^H-NMR (200 MHz, DMSO-*d_6_*, TMS) δ (ppm): 2.68 (t, 4H, *J* = 4.3 Hz, H2'' and H6''); 3.25 (t, 4H, *J* = 4.3 Hz, H3'' and H5''); 6.64 (s, 2H, NH_2_); 6.89 (d, 1H, *J* = 8.2 Hz, H6); 7.02 (s, 1H, H4); 7.62 (d, 1H, *J* = 8.2 Hz, H7); 7.73 (d, 2H, *J* = 8.6 Hz, H3′ and H5'); 7.87 (d, 2H, *J* = 8.6 Hz, H2' and H6'). ^13^C-NMR (50 MHz, DMSO-*d_6_*, TMS) δ (ppm): 26.5 (C3'' and C5''); 47.8 (C2'' and C6''); 107.2 (C-4); 116.1 (C-6); 117.4 (C-7a); 125.6 (C-7); 127.1 (C-3' and C-5'); 127.8 (C-2' and C-6'); 134.2 (C-4'); 134.5 (C-1'); 136.6 (C-3a); 155.5 (C-5); 166.3 (C-3); 166.8 (C-1). IR (υ_max_, KBr) υ (cm^−1^): 3464, 3365, 2918, 2856, 1768, 1710, 1637, 1379, 1164, 834. *Anal*. Calcd. for C_18_H_17_N_3_O_4_S_2_: C, 53.58; H, 4.25; N, 10.41. Found: C, 53.49; H, 4.19; N, 10.32.

*2-(4-(Thiomorpholinosulfonyl)phenyl)isoindolin-1-one* (LASSBio-577, **5**). In a 25 mL flask equipped with a reflux condenser, the reaction mixture containing the phthalimide derivative **1** (870 mg, 2.24 mmol), zinc dust (1.465 g, 22.4 mmol) and 20 mL of acetic acid was stirred and heated at 140 °C for 72 h. The end of the reaction was observed by TLC and the warm reaction medium was then filtered, followed by addition of distilled water to the filtrate until precipitation. Subsequently, the obtained precipitate was filtered through a Büchner funnel, giving the title compound as a white solid, 71% yield, mp > 250 °C. ^1^H-NMR (300 MHz, DMSO-*d_6_*, TMS) δ (ppm): 2.68 (t, 4H, *J* = 5.0 Hz, H2'' and H6''); 3.22 (t, 4H, *J* = 5.0 Hz, H3'' and H5''); 5.10 (s, 2H, H3); 7.55 (m, 1H, H4); 7.69 (m, 2H, H5 and H6); 7.81 (m, 3H, H7, H3' and H5'); 8.18 (d, 2H, *J* = 9.0 Hz, H2' and H6'). ^13^C-NMR (75 MHz, DMSO-*d_6_*, TMS) δ (ppm): 26.3 (C2'' and C6''); 47.7 (C3'' and C5''); 50.3 (C-3); 118.6 (C-3' and C-5'); 123.4 (C-7); 123.5 (C-6); 128.3 (C-4); 128.4 (C-2' and C-6'); 130.2 (C-7a); 131.7 (C-4'); 132.8 (C-5); 141.0 (C-1'); 143.4 (C-3a); 167.2 (C-1). IR (υ_max_, KBr) υ (cm^−1^): 2927, 2852, 1692, 1384, 1163, 843, 729. *Anal*. Calcd. for C_18_H_18_N_2_O_3_S_2_: C, 57.73; H, 4.84; N, 7.48. Found: C, 57.68; H, 4.81; N, 7.38.

*N-(4-(Thiomorpholinosulfonyl)phenyl)benzamide* (LASSBio-1446, **6**). Benzoyl chloride (**13**, 0.1 mL, 0.8 mmol) was slowly added to a solution of the key intermediate **8** (200 mg, 0.78 mmol) in 20 mL of methylene chloride. The reaction mixture was stirred for 20 min at room temperature and the end of the reaction was checked by TLC. The benzamide **6** was isolated by addition of 50 mL of methylene chloride and extraction, washing the organic layer with 10% aq HCl and brine. The organic layer was then dried over anhydrous Na_2_SO_4_, filtered and concentrated at reduced pressure to give the title compound as a white shiny powder, 76% yield, mp 204 °C. ^1^H-NMR (200 MHz, DMSO-*d_6_*, TMS) δ (ppm): 2.67 (t, 4H, *J* = 4.8 Hz, H2'' and H6''); 3.20 (t, 4H, *J* = 4.8 Hz, H3'' and H5''); 7.55 (m, 3H, H3, H4 and H5); 7.74 (d, 2H, *J* = 8.8 Hz, H3' and H5'); 7.97 (dd, 2H, *J* = 1.5 Hz and 8.0 Hz, H2 and H6); 8.07 (d, 2H, *J* = 8.8 Hz, H2' and H6'); 10.67 (s, 1H, CONH). ^13^C-NMR (50 MHz, DMSO-*d_6_*, TMS) δ (ppm): 26.4 (C2'' and C6''); 47.8 (C3'' and C5''); 120.0 (C-3 and C-5); 127.8 (C-3' and C-5'); 128.3 (C-2' and C-6'); 128.5 (C-2 and C-6); 130.2 (C-1); 132.0 (C-4); 134.4 (C-1'); 143.5 (C-4'); 166.2 (C=O). IR (υ_max_, KBr) υ (cm^−1^): 3381, 2916, 2858, 1683, 1336, 1160, 833, 733, 712. *Anal*. Calcd. for C_17_H_18_N_2_O_3_S_2_: C, 56.33; H, 5.01; N, 7.73. Found: C, 56.22; H, 4.88; N, 7.68.

#### 3.1.3. General Procedure for Synthesis of Carboxyamide *N*-Phenyl Sulfonamide Derivatives

The reaction mixture containing the phthalimide precursors (0.43 mmol), potassium hydroxide (49 mg, 0.86 mmol) and ethanol (5 mL) was vigorously stirred at room temperature for 1 hour and the end of the reaction was checked by TLC. The reaction medium was then diluted with 10 mL of methylene chloride followed by addition of 5 mL of distilled water. Subsequently, the extraction was performed in a separatory funnel. The aqueous layer was acidified with 10% aq HCl until pH 3 and the obtained precipitate was, then, filtered through a Büchner funnel to provide the carboxyamide derivatives in good yields.

*2-((4-(Thiomorpholinosulfonyl)phenyl)carbamoyl)benzoic acid* (LASSBio-596, **14**). The title compound was obtained from alkaline hydrolysis of the phthalimide precursor **1** as a white shiny powder, 86% yield, mp 187–189 °C. The melting point, ^1^H-NMR and IR data for compound **14** are in agreement with previous reports [8]. ^1^H-NMR (200 MHz, DMSO-*d_6_*, TMS) δ (ppm): 2.67 (m, 4H, H2'' and H6''); 3.18 (m, 4H, H3'' and H5''); 3.61 (br, 1H, COOH); 7.56 (m, 1H, H4); 7.60 (br, 1H, H3); 7.65 (dd, 1H, *J* = 1.7 Hz and 2.1 Hz, H5); 7.72 (d, 2H, *J* = 8.7 Hz, H2' and H6'); 7. 90 (d, 1H, *J* = 2.1 Hz, H6); 7.94 (d, 2H, *J* = 8.7 Hz, H3' and H5'); 10.82 (s, 1H, CONH). IR (υ_max_, KBr) υ (cm^−1^): 3495, 3325, 2929, 2857, 1731, 1693, 1330, 1159, 837, 724. *Anal*. Calcd. for C_18_H_18_N_2_O_5_S_2_.H_2_O: C, 50.93; H, 4.75; N, 6.60. Found: C, 51.08; H, 4.78; N, 6.59. 

*2,3,4,5-Tetrafluoro-6-((4-(thiomorpholinosulfonyl)phenyl) carbamoyl) benzoic acid* (LASSBio-1454, **15**). The title compound was obtained from alkaline hydrolysis of the phthalimide precursor **2e** as a white powder, 84% yield, mp > 250 °C. ^1^H-NMR (200 MHz, DMSO-*d_6_*, TMS) δ (ppm): 2.67 (br, 4H, H2'' and H6''); 3.19 (br, 4H, H3'' and H5''); 3.91 (br, 1H, COOH); 7.72 (d, 2H, *J* = 7.9 Hz, H2' and H6'); 7.85 (d, 2H, *J* = 7.9 Hz, H3' and H5'); 11.25 (s, 1H, CONH). IR (υ_max_, KBr) υ (cm^−1^): 3497, 3323, 2919, 2855, 1730, 1694, 1402, 1375, 1161, 838. *Anal*. Calcd. for C_18_H_14_F_4_N_2_O_5_S_2_: C, 45.19; H, 2.95; N, 5.86. Found: C, 45.23; H, 3.01; N, 5.81.

### 3.2. Chemical Hydrolysis and Plasma Stability Studies

The compounds (5 µM) were incubated at 37 °C under stirring in 250 µL of PBS (pH 7.4) or rat plasma (50% in PBS) for different times (0, 2.5, 5, 10, 30 and 60 min). The reaction was stopped by addition of 750 µL of acetonitrile (0.4% trifluoroacetic acid). The samples were centrifuged and the supernatants were filtered and analyzed by HPLC. The HPLC analyses were conducted in a Shimadzu LC-20AD apparatus equipped with a SHIM-PACK CLC-ODS analytical column (4.6 mm × 250 mm) or a Kromasil 100-5C18 (4.6 mm × 250 mm) and with a Shimadzu SPD-M20A diode array detector. The mobile phase was consisted of acetonitrile-water (60:40% v/v), both acidified with 0.1% trifluoroacetic acid, and the flow rate was 1 mL/min. This method allowed monitoring the consumption of the phthalimide precursors LASSBio-468 (**1**) and LASSBio-1439 (**2e**) and the appearance of the corresponding metabolites LASSBio-596 (**14**) and LASSBio-1454 (**15**), respectively.

### 3.3. Biological Assays

#### 3.3.1. Animals

A/J and Swiss-Webster mice (18–20 g) were obtained from the Oswaldo Cruz Foundation breeding colonies (Rio de Janeiro, Brazil). They were kept in the animal-housing facilities at a controlled room temperature (22–25 °C) and a 12h/12h light:dark cycle (6 a.m. to 6 p.m.). All the procedures related to care and use of laboratory animals in this study were examined and approved by the Animal Ethics Committee of the Oswaldo Cruz Foundation (CEUA-FIOCRUZ, license 034/09).

#### 3.3.2. Model of Lung Inflammation Induced by LPS and Treatment

Lung inflammation was induced in anesthetized A/J mice by an intranasal instillation of LPS (from *E.coli* serotype 0127:B8; SIGMA, St. Louis, MO, USA) (1 mg/mL) or saline (control group). The testing compounds were dissolved in saline solution containing 0.5% Tween 80 and administered orally or intraperitoneally (50 or 100 mg/kg) 1 h before LPS instillation. All solutions were prepared immediately before use. The evaluation was conducted 24 h after LPS instillation.

#### 3.3.3. Assessment of Respiratory Mechanics

Airway responsiveness was assessed as a change in airway function after challenge with aerosolized methacholine (3, 9 and 27 mg/mL) in a Buxco System (Finepoint). Mice were anesthetized with nembutal (60 mg/kg) and neuromuscular activity was blocked with bromide pancuronium (1 mg/kg). Tracheostomized mice were mechanically ventilated and the lung function was assessed. The trachea was cannulated and the cannula connected to a pneumotachograph. The air flow and the transpulmonary pressure were recorded using a Buxco pulmonary mechanics processing system (Finepoint). The parameters of resistance (R_L_) (cmH_2_O/mL/s) and dynamic lung elastance (mL/cmH_2_O) in each breath cycle were calculated. Lung elastance was calculated as the inverse of compliance values. Analogical signals from the computer were digitized using a Buxco Analog/Digital Converter (Buxco Electronics). Increasing concentrations of methacholine (3, 9 and 27 mg/mL) were aerosolized for 5 min each and basal lung resistance and lung function were assessed under condition of phosphate buffered saline (PBS) aerosolization.

#### 3.3.4. Histopathological Analysis

Immediately after lung function analysis, the animals were killed and the lung was perfused with saline/EDTA solution throughout the heart. Lungs were removed and the left lobe was fixed with 4% formaldehyde and embedded in paraffin. 5 μm-thick slices were obtained by using a microtome and stained with Picrus-sirius. The material was examined using an optical microscope (200×). The right lung lobes were quick-frozen by immersion in liquid nitrogen.

#### 3.3.5. Tissue Myeloperoxidase Quantification

Lung samples were homogenized in 5 μM EDTA buffer solution containing 0.5% hexadecyl-trimethylammonium bromide (HTBA; SIGMA, St. Louis, MO, USA) using a tissue homogenizer. Samples were centrifuged at 1,700 × g for 15 min at 4 °C. The supernatants were collected, centrifuged at 15,295 × g for 15 min at 4 °C and assayed spectrophotometrically for MPO determination. 50 μL of sample were mixed with 50 μL of a EDTA/HTBA solution containing 0.68 mg/mL of *o*-dianisidine dihydrochloride (SIGMA) and 0.0005% hydrogen peroxide. The absorbance was read at 460 nm using a spectrophotometer and the results were expressed as optical density.

#### 3.3.6. Peritoneal Macrophages Isolation 

Swiss-Webster mice were killed by CO_2_ asphyxiation and peritoneal macrophages obtained by washing the peritoneal cavity with 10 μM EDTA solution. The cells were pelleted (433 × g, 5 min) and ressuspended in RPMI 1640 Medium (Invitrogen Life Technologies, Grand Island, NY, USA). Total mononuclear cells counts were performed in Neubauer chamber after sample dilution in Turk’s solution (acetic acid 0.2%). The cells were added (0.5 × 106 cells/mL) to a 24-well plate and incubated (37 °C and 5% CO_2_) in RPMI 1640 medium with bovine fetal serum (BSA) (2%) (Cultilab, Campinas, São Paulo, Brazil) supplemented with 200 U/mL penicillin and 200 mg/mL streptomycin for adhesion (1 h). After the adhesion step, the medium was removed and the macrophages were incubated in RPMI 1640 medium without BSA and supplemented with 200 U/mL penicillin and 200 mg/mL streptomycin. The cells were incubated with the testing compounds (100 μM) at 37 °C and 5% CO_2_ for 1 h and then stimulated with LPS (0.1 μg/mL). Twenty four hours later, the cells were centrifuged (433 × g, 5 min) and the supernatants were recovered and frozen at -80°C for further cytokine quantification by ELISA.

#### 3.3.7. ELISA

Levels of TNF-α in macrophage culture supernatants and lung tissue samples were measured by commercial ELISA kits (R&D Systems Inc, Minneapolis, MN, USA) according to manufacturer’s protocols. 

#### 3.3.8. Statistical Analysis

The statistical analysis was performed with ANOVA followed by the Newman-Keuls Student test or with Student’s t-test.

## 4. Conclusions 

Among the synthesized phenyl sulfonamide derivatives **2a**–**h** and **3**–**8**, designed by structural modification on the anti-inflammatory lead-compound LASSBio-468 (**1**), the tetrafluorophthalimide analogue LASSBio-1439 (**2e**) stands out showing an anti-TNF-α effect *in vitro* similar to the standard thalidomide (**9**). These results confirm the data previously described in the literature [11,13,25,26] suggesting the relevance of tetrafluorination of the phthalimide moiety to improve the anti-TNF-α activity in comparison with the non-fluorinated precursor.

The relevance of tetrafluorination in the phthalimide nucleus was also demonstrated by the anti-inflammatory profile of **2e** after oral administration in a murine model of pulmonary inflammation. Moreover, this functionalization increased the chemical and enzymatic lability of the phthalimide subunit, indicating that the anti-inflammatory and anti-TNF-α effects observed for **2e** *in vivo* are probably due to the corresponding carboxamide metabolite LASSBio-1454 (**15**).

The tetrafluorinated carboxamide metabolite **15** has shown an anti-TNF-α effect *in vitro* similar to that observed for the phthalimide precursor **2e**. Additionally, the tetrafluorometabolite **15** presented pronounced anti-inflammatory effects *in vivo* after intraperitoneal administration; and a higher inhibitory effect in the control of neutrophils infiltration into the lung tissue when compared with the non-fluorinated analogue **14**, showing once again the relevance of this functionalization.

## Figures and Tables

*Reagents and conditions*: (**a**) acetic anhydride, glacial acetic acid, CH_3_CO_2_Na anhydrous, r.t., 30 min, 90%; (**b**) HSO_3_Cl, 60 °C, 30 min, 85%; (**c**) thiomorpholine, CH_2_Cl_2_, r.t., 30 min, 65%; (**d**) KOH, H_2_O, MeOH, 60 °C, 3 h, 93%.

*Reagents and conditions*: (**e**) glacial acetic acid, 140 °C, 3 h, 73%; (**f**) solvent free, 180 °C, 1–4 h, 50–95%.

*Reagents and conditions*: (**g**) Fe°, NH_4_Cl, EtOH, H_2_O, 78 °C, 2 h, 57–61%.

*Reagents and conditions*: (**h**) Zn dust, glacial acetic acid, 140 °C, 72 h, 71%.

*Reagents and conditions*: (**i**) CH_2_Cl_2_, r.t, 20 min, 76%.

*Reagents and conditions*: (**j**) KOH, EtOH, r.t, 1 h, 84–86%.

## References

[1] Moldoveanu B., Otmishi P., Jani P., Walker J., Sarmiento X., Guardiola J., Saad M., Yu J. (2009). Inflammatory mechanisms in the lung. J. Inflamm. Res..

[2] Medzhitov R. (2010). Inflammation 2010: New adventures of an old flame. Cell.

[3] Dai J., Liu B., Li Z. (2009). Regulatory T cells and Toll-like receptors: What is the missing link?. Int. Immunopharmacol..

[4] Mukhopadhyay S., Hoidal J.R., Mukherjee T.K. (2006). Role of TNFα in pulmonary pathophysiology. Respir. Res..

[5] Wheeler A.P., Bernard G.R. (2007). Acute lung injury and the acute respiratory distress syndrome: A clinical review. Lancet.

[6] Hashimoto Y. (2002). Structural development of biological response modifiers based on thalidomide. Bioorg. Med. Chem..

[7] Barbosa M.L.C., Fumian M.M., Miranda A.L.P., Barreiro E.J., Lima L.M. (2011). Therapeutic approaches for tumor necrosis factor inhibition. Braz. J. Pharm. Sci..

[8] Lima L.M., Castro P., Machado A.L., Fraga C.A., Lugnier C., de Moraes V.L., Barreiro E.J. (2002). Synthesis and anti-inflammatory activity of phthalimide derivatives, designed as new thalidomide analogues. Bioorg. Med. Chem..

[9] Alexandre-Moreira M.S., Takiya C.M., de Arruda L.B., Pascarelli B., Gomes R.N., Castro Faria Neto H.C., Lima L.M., Barreiro E.J. (2005). LASSBio-468: A new achiral thalidomide analogue which modulates TNF-alpha and NO production and inhibits endotoxic shock and arthritis in an animal model. Int. Immunopharmacol..

[10] Lima L.M., Manssour Fraga C.A., Gonçalvez Koatz V.L., Barreiro E.J. (2006). Thalidomide and analogs as anti-inflammatory and immunomodulator drug candidates. Anti-Inflamm. Anti-Allergy Agents Med. Chem..

[11] Niwayama S., Turk B.E., Liu J.O. (1996). Potent inhibition of tumor necrosis factor-alpha production by tetrafluorothalidomide and tetrafluorophthalimides. J. Med. Chem..

[12] Muller G.W., Chen R., Huang S.Y., Corral L.G., Wong L.M., Patterson R.T., Chen Y., Kaplan G., Stirling D.I. (1999). Amino-substituted thalidomide analogs: Potent inhibitors of TNF-alpha production. Bioorg. Med. Chem. Lett..

[13] Gütschow M., Hecker T., Thiele A., Hauschildt S., Eger K. (2001). Aza analogues of Thalidomide: Synthesis and evaluation as inhibitors of tumor necrosis factor-α production *in vitro*. Bioorg. Med. Chem..

[14] Bastos R.S., da Cunha A.S., da Silva L.C., de Oliveira C.C.P., Rezende C.M., Pinto A.C. (2008). Preparo da *para*-cloroanilina: Um experimento simples, rápido e barato. Quim. Nova.

[15] Barbosa M.L.C., Melo G.M.A., Cupertino da Silva Y.K., Lopes R.O., de Souza E.T., de Queiroz A.C., Smaniotto S., Alexandre-Moreira M.S., Barreiro E.J., Lima L.M. (2009). Synthesis and pharmacological evaluation of *N*-phenyl-acetamide sulfonamides designed as novel non-hepatotoxic analgesic candidates. Eur. J. Med. Chem..

[16] Stewart J. (1922). Sulfonation of acetanilide. J. Chem. Soc..

[17] Furniss B.S., Hannaford A.J., Rogers V., Smith P.W.G., Tatchell A.R. (1978). Vogel’s Textbook of Practical Organic Chemistry.

[18] Cremlyn R.J., Swinbourne F.J., Nunes R.J. (1985). Phthalimidobenzenesulphonyl derivatives. Quim. Nova.

[19] Almansa C., Alfón J., de Arriba A.F., Cavalcanti F.L., Escamilla I., Gómez L.A., Miralles A., Soliva R., Bartrolí J., Carceller E. (2003). Synthesis and structure-activity relationship of a new series of COX-2 selective inhibitors: 1,5-diarylimidazoles. J. Med. Chem..

[20] Capitosti S.M., Hansen T.P., Brown M.L. (2004). Thalidomide analogues demonstrate dual inhibition of both angiogenesis and prostate cancer. Bioorg. Med. Chem..

[21] Trujillo-Ferrara J., Correa-Basurto J., Espinosa J., García J., Martínez F., Miranda R. (2005). Solvent-free synthesis of arylamides and arylimides, analogues of acetylcholine. Synth. Commun..

[22] Julémont F., de Leval X., Michaux C., Damas J., Charlier C., Durant F., Pirotte B., Dogné J.-M. (2002). Spectral and crystallographic study of pyridinic analogues of nimesulide: Determination of the active form of methanesulfonamides as COX-2 selective inhibitors. J. Med. Chem..

[23] Brewster J.H., Fusco A.M., Carosino L.E., Corman B.G. (1963). Reduction of phthalic acid and its derivatives by zinc. J. Org. Chem..

[24] Eisch J.J., Sanchez R. (1986). Selective, oxophilic imination of ketones with bis(dichloroaluminum) phenylimide. J. Org. Chem..

[25] Hashimoto Y. (1998). Novel biological response modifiers derived from thalidomide. Curr. Med. Chem..

[26] Collin X., Robert J.-M., Wielgosz G., Le Baut G., Bobin-Dubigeon C., Grimaud N., Petit J.-Y. (2001). New anti-inflammatory *N*-pyridinyl(alkyl)phthalimides acting as tumour necrosis factor-α production inhibitors. Eur. J. Med. Chem..

[27] Schumacher H., Smith R.L., Williams R.T. (1965). The metabolism of thalidomide: the spontaneous hydrolysis of thalidomide in solution. Br. J. Pharmacol. Chemother..

[28] Melchert M., List A. (2007). The thalidomide saga. Int. J. Biochem. Cell Biol..

[29] Rocco P.R.M., Xisto D.G., Silva J.D., Diniz M.F.F.M., Almeida R.N., Luciano M.N., Medeiros I.A., Cavalcanti B.C., Ferreira J.R.O., de Moraes M.O. (2010). LASSBio-596: da descoberta aos ensaios pré-clínicos. Rev. Virtual Quim..

[30] Lee Y.M., Moon M.U., Vajpayee V., Filimonov V.D., Chi K.W. (2010). Efficient and economic halogenation of aryl amines via arenediazonium tosylate salts. Tetrahedron.

[31] Cragoe E.J., Hamilton C.S. (1945). The synthesis of arsenicals containing certain heterocyclic Nuclei. J. Am. Chem. Soc..

[32] Pons M., Feliz M., Giralt E. (1985). 13C-NMR spectra of fluorinated molecules using 19F-13C polarization transfer. Tetrahedron Lett..

